# Mental Health and Well-Being in the Modern Era: A Comprehensive Review of Challenges and Interventions

**DOI:** 10.7759/cureus.77683

**Published:** 2025-01-19

**Authors:** Aminat Magomedova, Ghizal Fatima

**Affiliations:** 1 Department of Population, Lomonosov Moscow State University, Moscow, RUS; 2 Department of Public Health, Era's Lucknow Medical College and Hospital, Lucknow, IND

**Keywords:** digital health, interventions, mental disorders, mental health, modern challenges, well-being

## Abstract

A critical global concern in the modern era is mental health and well-being, where rapid socioeconomic change, technological advancements, and lifestyle shifts have significantly impacted individuals' psychological health. Primary stressors today include urbanization, digital dependency, social isolation, and economic pressures, alongside the escalating prevalence of mental health disorders such as depression, anxiety, and burnout. The COVID-19 pandemic has further exacerbated global mental health crises, increasing the vulnerability of populations during times of uncertainty and instability. This paper critically examines emerging connections between mental health and lifestyle factors such as sleep, diet, and exercise. Interventions are discussed from a multidimensional perspective, encompassing pharmacological treatments, psychotherapy, digital mental health tools, and community-based programs. Special attention is given to the rise of telemedicine and mobile mental health apps, offering innovative solutions to bridge gaps in mental healthcare accessibility. Furthermore, the review underscores the importance of preventive approaches, promoting mental health literacy, reducing stigma, and fostering resilience through mindfulness, cognitive behavioral techniques, and social support systems.

## Introduction and background

In the modern era, mental health and well-being have become pivotal aspects of global health, gaining increasing recognition as essential components of overall well-being. Mental health refers to a state of emotional, psychological, and social well-being, enabling individuals to cope with stress, work productively, and contribute to their community. Mental well-being, a broader term, encompasses the positive aspects of mental health, including resilience, life satisfaction, and a sense of purpose. Mental health is no longer viewed merely as the absence of mental illness but as a holistic state that encompasses emotional, psychological, and social well-being, influencing how individuals think, feel, and behave. It affects daily life, relationships, work productivity, and overall quality of life. However, the fast-paced nature of contemporary society, marked by rapid technological advancements, social and economic pressures, and lifestyle shifts, has given rise to new challenges for maintaining mental health and well-being [1].

The prevalence of mental health disorders such as depression, anxiety, and stress-related conditions has surged in recent decades, with the World Health Organization (WHO) estimating that approximately one in four people will be affected by mental health issues at some point in their lives [2]. The global mental health crisis is underscored by alarming statistics, with the WHO estimating that nearly 800,000 people die by suicide each year, representing one person every 40 seconds. Countries such as India and the United States have seen rising suicide rates, highlighting the urgent need for targeted mental health interventions and support systems [2]. Urbanization, increased screen time, social media exposure, and the erosion of traditional social support systems have significantly altered the mental landscape. Prolonged work hours, economic insecurity, and societal pressures to constantly perform and achieve have further exacerbated these issues, leading to burnout, chronic stress, and widespread mental fatigue. The onset of the COVID-19 pandemic in 2020 has compounded these challenges, bringing with it not only a physical health crisis but also a parallel mental health crisis. Social isolation, fear of illness, financial instability, and uncertainty about the future have led to a sharp increase in the prevalence of mental health issues. Studies have shown a dramatic rise in anxiety, depression, and post-traumatic stress disorder (PTSD) during and after the pandemic, highlighting the need for enhanced mental health care and intervention strategies [3].

Mental health and well-being are shaped by a complex interplay of biological, psychological, and environmental factors. Biologically, mental health can be influenced by genetics, neurochemical imbalances, and underlying medical conditions. The role of neurobiology, particularly the balance of neurotransmitters such as serotonin, dopamine, and cortisol, is crucial in understanding mood regulation, stress responses, and the development of mental health disorders. Moreover, research points to the gut-brain axis as a significant player in mental health, linking gut microbiota imbalances with conditions such as depression and anxiety [4]. Psychological factors such as trauma, personality traits, cognitive patterns, and emotional resilience also contribute to an individual's mental health status. The ability to cope with stress, process emotions, and engage in healthy relationships plays a critical role in maintaining mental well-being. Furthermore, environmental determinants, including socioeconomic status, living conditions, education, and access to healthcare, are powerful influences on mental health. People in disadvantaged or marginalized communities are often at higher risk of developing mental health issues due to the compounded effects of poverty, discrimination, and lack of resources [5,6].

In response to these rising challenges, this review sets out to achieve three key objectives: (1) identify the key challenges contributing to the mental health crisis in the modern era, focusing on the interplay of biological, psychological, and environmental factors; (2) evaluate the effectiveness of existing interventions, including pharmacological treatments, psychological therapies, and digital mental health tools, in addressing mental health challenges; and (3) propose future research directions aimed at developing adaptive, personalized, and integrative strategies to promote mental well-being in a rapidly evolving world.

By examining these objectives, this review aims to provide a comprehensive understanding of the complexities surrounding mental health and well-being while offering actionable insights to address the pressing mental health challenges. For this to be clear, we have added details on search strategies, inclusion criteria, risk of bias assessment, and summary methods to ensure replicability and reproducibility.

Search strategies

The search strategy involved a comprehensive review of multiple databases, including PubMed, Scopus, and Web of Science, using relevant keywords such as "mental health", "suicide rates", "screen time and mental health", "sleep and cognitive function", and "lifestyle factors in mental health". Additionally, filters were applied to refine the search for peer-reviewed articles published in the last 10 years.

Inclusion criteria

Studies were included if they met the following criteria: (1) focused on mental health and its associated factors (e.g., suicide rates, screen time, sleep); (2) published in peer-reviewed journals; and (3) available in English.

## Review

Overview of mental health and well-being in the modern era

Mental health and well-being are increasingly recognized as essential components of overall health in the modern era. Defined by the WHO as a state of mental, emotional, and social well-being, mental health influences how individuals handle stress, make choices, and relate to others. However, rapid social, economic, and technological changes have dramatically reshaped the landscape of mental health. Factors such as urbanization, economic pressures, increased screen time, and social isolation contribute to the rising prevalence of mental health disorders such as anxiety, depression, and stress-related conditions. According to WHO estimates, approximately one billion people globally are affected by mental health disorders, with depression being a leading cause of disability. Additionally, the COVID-19 pandemic has exacerbated these issues, causing significant disruptions to daily life, financial instability, and heightened uncertainty, leading to a surge in anxiety and depression [2].

The role of digital tools in mental health care, such as teletherapy, mental health apps, and online support platforms, has become increasingly popular, especially in post-COVID patients. The pandemic has significantly exacerbated post-COVID mental health challenges, leading to heightened levels of anxiety, depression, and PTSD, particularly among frontline workers and individuals facing prolonged isolation [3]. Additionally, social determinants such as income inequality, access to healthcare, and education continue to play a critical role in mental health outcomes, with marginalized communities experiencing disproportionate effects. Advancements in personalized approaches to mental health treatment, driven by insights from genetics and neurobiology, gained attention for their potential to offer more tailored interventions. Furthermore, lifestyle factors, including sleep, diet, exercise, and substance use, have been increasingly recognized for their influence on mental health, emphasizing the need for a holistic approach to care. Lastly, youth mental health has emerged as a growing concern, with a surge in mental health issues among adolescents, largely due to social media, academic pressures, and the broader impact of the COVID-19 pandemic. Including these recent findings will provide a more complete picture of the current mental health landscape, guiding future research and informing more effective interventions [4]. The modern era's focus on productivity, societal expectations, and technological dependence further impacts mental well-being, as individuals often face chronic stress and burnout. Addressing these growing mental health challenges requires multidimensional approaches that incorporate preventive strategies, mental health literacy, and accessible interventions to promote holistic well-being in today's fast-paced world [7].

The modern mental health crisis

Rising Prevalence of Mental Health Disorders (Depression, Anxiety, and Burnout)

The prevalence of mental health disorders, such as depression, anxiety, and burnout, has been steadily increasing in recent decades, posing significant public health challenges. Depression, identified by the WHO as a leading cause of disability worldwide, affects more than 300 million people globally. Depression is classified based on the criteria outlined in the International Classification of Diseases (ICD) and the Diagnostic and Statistical Manual of Mental Health Disorders (DSM-5). According to ICD-10, a diagnosis requires at least four symptoms, including two core symptoms: depressed mood, loss of interest or pleasure, and reduced energy persisting for at least two weeks. The DSM-5 specifies that a diagnosis of major depressive disorder (MDD) requires at least five symptoms, with at least one being a core symptom (depressed mood or loss of interest), lasting for a minimum of two weeks and causing significant distress or functional impairment. Chronic depression, termed persistent depressive disorder (dysthymia) in DSM-5, is characterized by a depressed mood most of the day, more days than not, for at least two years (one year for children/adolescents), accompanied by at least two additional symptoms such as low energy, poor concentration, or feelings of hopelessness. In both ICD and DSM-5, chronic depression is diagnosed when symptoms persist without a symptom-free period exceeding two months. These criteria ensure consistency in identifying and managing depression across clinical settings [3,4].

Anxiety disorders, which include generalized anxiety disorder (GAD), social anxiety, and panic disorder, impact nearly 264 million people, manifesting as excessive worry, fear, and avoidance behaviors. Anxiety-related disorders are classified based on specific criteria outlined in the ICD and DSM-5, each specifying distinct symptoms and timeframes for diagnosis. GAD, according to DSM-5, requires excessive worry and anxiety about various events or activities occurring more days than not for at least six months, accompanied by at least three symptoms such as restlessness, fatigue, difficulty concentrating, irritability, muscle tension, or sleep disturbances. The ICD-10 criteria are similar but emphasize pervasive anxiety not confined to specific situations.

Panic disorder involves recurrent, unexpected panic attacks with at least one month of persistent concern about having more attacks or their consequences, as per DSM-5 and ICD-10. Social anxiety disorder is characterized by a marked fear or anxiety about social situations where scrutiny by others is possible, persisting for at least six months, with avoidance or distress significantly impairing daily functioning. Specific phobias involve an intense, irrational fear of specific objects or situations lasting six months or more, with avoidance behaviors. Separation anxiety disorder, typically diagnosed in children but also applicable to adults, requires excessive fear of separation from attachment figures for at least four weeks in children and six months in adults. Providing these timeframes and symptom criteria enhances the understanding of anxiety-related disorders, highlighting the nuances between different conditions.

Burnout, a state of emotional, physical, and mental exhaustion caused by prolonged stress, particularly in the workplace, has gained recognition as a widespread issue [2,3]. Defined by emotional fatigue, detachment, and a sense of reduced accomplishment, burnout is particularly prevalent in high-pressure industries such as healthcare, education, and corporate sectors. The WHO officially classified burnout as an occupational phenomenon in 2019, further highlighting its growing significance. Factors such as increased work demands, economic pressures, social isolation, and lifestyle changes contribute to the rising incidence of these disorders. The COVID-19 pandemic has also intensified mental health issues, with reports of depression and anxiety surging due to prolonged lockdowns, job losses, and uncertainty about the future [2].

Impact of Urbanization, Digital Dependency, and Social Isolation

Urbanization, digital dependency, and social isolation have profoundly impacted mental health, contributing to the rising prevalence of mental health disorders such as anxiety, depression, and loneliness in modern society. Urbanization often brings economic opportunities, but it also introduces challenges such as overcrowding, pollution, noise, and a fast-paced lifestyle, all of which can exacerbate stress and mental fatigue. Urban residents face higher rates of mental health disorders compared to those in rural areas, with research indicating that living in cities increases the risk of anxiety and mood disorders by 21% and 39%, respectively. The breakdown of community ties and the isolation often felt in densely populated areas further heighten these risks. Digital dependency, driven by the widespread use of smartphones, social media, and online platforms, has reshaped human interactions and daily routines. Excessive screen time, particularly on social media, is linked to increased feelings of inadequacy, anxiety, and depression, especially in younger populations. Constant connectivity can lead to information overload, disturbed sleep, and a lack of real-world social connections, contributing to poor mental health [8]. Whether due to urban living or excessive digital engagement, it leads to feelings of loneliness and alienation. Studies have shown that prolonged social isolation can increase the risk of depression, anxiety, and cognitive decline, emphasizing the need for stronger social support systems in modern societies [8].

Mental Health During and After the COVID-19 Pandemic

The COVID-19 pandemic has had a profound impact on mental health globally, exacerbating existing mental health issues and creating new challenges. During the pandemic, widespread lockdowns, social distancing, and economic uncertainty contributed to an unprecedented surge in mental health disorders such as anxiety, depression, and PTSD [9]. The WHO reported a 25% increase in anxiety and depression cases globally during the first year of the pandemic [2]. Fear of infection, grief from losing loved ones, financial instability, and prolonged isolation were key factors driving this increase. Healthcare workers, frontline employees, and individuals with pre-existing mental health conditions were particularly vulnerable to psychological stress and burnout. Increased workloads, emotional exhaustion, and the trauma of witnessing high mortality rates contributed to a marked rise in mental health issues among these populations. Moreover, the pandemic's disruption of mental health services limited access to care, compounding the situation for many individuals. As the pandemic subsided, post-COVID mental health challenges emerged, with many experiencing what is termed "pandemic fatigue," chronic stress, exhaustion, and emotional burnout. Additionally, long COVID has been linked to neurological and psychological symptoms such as brain fog, anxiety, and depression. Addressing these lingering mental health challenges requires long-term interventions, greater accessibility to care, and sustained efforts to build psychological resilience in the aftermath of the pandemic [10].

Biological determinants of mental health

Neurobiology of Mental Health: Neurotransmitters and Brain Regions Involved

The neurobiology of mental health is intricately linked to the regulation of neurotransmitters and the functioning of specific brain regions. Neurotransmitters, chemical messengers in the brain, play a crucial role in mood regulation, emotional processing, and mental well-being. Dysregulation in neurotransmitter systems is often associated with mental health disorders such as depression, anxiety, and schizophrenia.

Key Neurotransmitters Involved in Mental Health

Serotonin: Serotonin, often called the "feel-good" neurotransmitter, plays a crucial role in mental health by regulating mood, emotions, and behavior. Synthesized from the amino acid tryptophan, serotonin influences various brain functions, including sleep, appetite, and stress response. Its imbalance is closely linked to mental health disorders such as depression, anxiety, and obsessive-compulsive disorder (OCD). Low serotonin levels are often associated with feelings of sadness, irritability, and a reduced ability to handle stress. Many antidepressant medications, such as selective serotonin reuptake inhibitors (SSRIs), work by increasing serotonin availability in the brain, thereby alleviating symptoms of these disorders. Beyond mood, serotonin impacts cognitive functions such as memory and decision-making, as well as social behaviors. Factors such as diet, exercise, sunlight exposure, and gut health significantly influence serotonin levels. A holistic approach that combines pharmacological treatments, psychotherapy, and lifestyle modifications can effectively enhance serotonin balance, promoting mental well-being and resilience [11].

Dopamine: Dopamine, a key neurotransmitter in the brain, plays a vital role in mental health and well-being by regulating reward, motivation, and pleasure. It drives goal-directed behavior, reinforcing positive actions and fostering feelings of satisfaction. Imbalances in dopamine levels are linked to various mental health conditions, such as depression, schizophrenia, and attention-deficit/hyperactivity disorder (ADHD). Dopamine synthesis primarily occurs in the substantia nigra and ventral tegmental area (VTA) of the brain. Dopamine-enhancing medications, such as D2 receptor agonists (e.g., pramipexole, ropinirole), act by stimulating dopamine receptors, mimicking dopamine's effects. These medications are commonly used in mood disorders such as depression and bipolar disorder, where they help improve motivation, reward processing, and mood regulation. By enhancing dopaminergic activity, they address symptoms related to anhedonia and low energy, often seen in these conditions. Parkinson's disease is a neurodegenerative disorder associated with low dopamine levels due to the degeneration of dopaminergic neurons in the substantia nigra, leading to motor and non-motor symptoms [12]. Low dopamine activity can result in reduced motivation, apathy, and difficulty experiencing pleasure, often seen in depression and anhedonia. Conversely, excessive dopamine activity in certain brain regions is associated with symptoms of psychosis and addiction. Dopamine also influences cognitive functions such as focus, memory, and decision-making, which are crucial for everyday functioning. Factors such as physical activity, a balanced diet, and engaging in rewarding activities can naturally enhance dopamine levels. Understanding and optimizing dopamine pathways through medication, therapy, and lifestyle changes are essential for promoting mental well-being and resilience [12].

Norepinephrine: Norepinephrine, a neurotransmitter and stress hormone, plays a critical role in health and well-being by regulating alertness, attention, and the body's response to stress. Norepinephrine is synthesized primarily in the locus coeruleus of the brainstem and produced by the adrenal medulla in the peripheral nervous system. It is a key component of the "fight or flight" response, preparing the body to react to challenges by increasing heart rate, blood pressure, and energy availability. In mental health, norepinephrine influences mood, motivation, and cognitive functions such as focus and decision-making. Imbalances in norepinephrine levels are linked to conditions such as depression, anxiety, and ADHD. Low levels can lead to fatigue, poor concentration, and apathy, while excessive levels may contribute to heightened stress, irritability, and insomnia. Medications such as serotonin-norepinephrine reuptake inhibitors (SNRIs) target norepinephrine pathways to treat depression and anxiety disorders. Lifestyle factors such as regular exercise, stress management, and adequate sleep can naturally support norepinephrine balance, promoting mental clarity, emotional stability, and overall resilience [13].

GABA (gamma-aminobutyric acid): GABA is a primary inhibitory neurotransmitter in the brain, crucial for maintaining mental health and emotional well-being. By reducing neuronal excitability, GABA acts as a calming agent, helping to regulate anxiety, stress, and overall brain activity. It counterbalances excitatory neurotransmitters such as glutamate, ensuring that the brain does not become overstimulated, essential for maintaining mental equilibrium. Low levels of GABA are associated with mental health disorders such as anxiety, depression, and insomnia. Low levels of GABA, which is an inhibitory neurotransmitter, contribute to depression by reducing the brain's ability to regulate excitatory signals, leading to heightened neural activity and stress responses. This imbalance increases susceptibility to overactivation of the hypothalamic-pituitary-adrenal (HPA) axis, resulting in elevated cortisol levels, which are strongly associated with depression. Additionally, GABA deficiency impairs synaptic plasticity and emotional regulation in key brain regions such as the prefrontal cortex and amygdala, further exacerbating depressive symptoms. People with GAD or panic disorder often exhibit impaired GABA functioning, leading to heightened states of worry and tension. Similarly, GABA deficiencies can contribute to sleep disturbances and mood swings, exacerbating conditions such as bipolar disorder and MDD.

Pharmacological interventions, such as benzodiazepines, target GABA receptors to enhance their calming effects, providing relief from anxiety and insomnia. Non-pharmacological approaches, including mindfulness, yoga, and deep-breathing exercises, can naturally boost GABA activity. A balanced diet rich in magnesium, zinc, and B vitamins also supports GABA production. Foods rich in GABA include fermented products such as kimchi, miso, and yogurt, which naturally boost GABA levels. Whole grains such as brown rice, oats, and barley are also excellent sources, along with nuts and seeds like almonds, walnuts, and sunflower seeds. Vegetables such as spinach, broccoli, and tomatoes, as well as fruits like bananas and citrus fruits, further support GABA production. Incorporating these foods into the diet can help promote relaxation and improve mental well-being. Understanding GABA's role highlights its importance in creating a balanced neural environment. By modulating GABA levels through lifestyle changes, therapy, or medication, individuals can achieve better mental health and resilience against stress [14].

Glutamate: Glutamate is the brain's primary excitatory neurotransmitter, playing a crucial role in mental health and overall brain function. It is essential for cognitive processes such as learning, memory, and neural plasticity, as it facilitates the transmission of signals between neurons. However, maintaining a balance in glutamate levels is vital, as both deficiencies and excesses can have significant mental health implications. Excessive glutamate activity, often referred to as excitotoxicity, can lead to neuronal damage and is implicated in conditions such as depression, anxiety, schizophrenia, and neurodegenerative diseases such as Alzheimer's and Parkinson's. On the other hand, insufficient glutamate activity can impair cognitive functions and contribute to mood disorders. Glutamate dysregulation is also linked to addiction and bipolar disorder, where shifts in excitatory signaling may exacerbate symptoms. Medications such as NMDA (N-methyl-D-aspartate) receptor antagonists, such as ketamine, have shown promise in rapidly alleviating treatment-resistant depression by modulating glutamate pathways. Lifestyle factors, including stress management, a balanced diet, and regular exercise, can help regulate glutamate levels. Emerging research into dietary interventions and supplements, such as magnesium and omega-3 fatty acids, highlights their potential role in supporting glutamate balance. Understanding and targeting glutamate pathways is critical for advancing mental health treatments and promoting cognitive well-being [15].

Brain Regions Involved in Mental Health

Mental health is intricately linked to the functioning of specific brain regions, each playing a critical role in regulating emotions, behaviors, and cognitive processes. The prefrontal cortex, responsible for executive functions, decision-making, and emotional regulation, often shows reduced activity in individuals with depression, contributing to difficulties in managing emotions and maintaining focus. Dysregulation in this area is also linked to anxiety disorders. The amygdala, which processes emotions such as fear and pleasure, is hyperactive in anxiety-related conditions such as GAD, PTSD, and panic disorders, leading to heightened emotional responses and fear processing. The hippocampus, crucial for memory formation and emotional regulation, is particularly vulnerable to chronic stress and depression, which can reduce its volume and impair both memory and emotional control. Interestingly, antidepressant treatments have been shown to stimulate neurogenesis in the hippocampus, aiding recovery. The hypothalamus plays a central role in regulating the body's stress response through the HPA axis. Several areas of the hypothalamus are crucial in regulating mental health. The paraventricular nucleus (PVN) plays a key role in controlling the HPA axis, which is involved in stress responses and is linked to mood disorders such as depression and anxiety. The supraoptic nucleus (SON) regulates vasopressin, influencing social behavior and stress reactions. The arcuate nucleus affects appetite, reward, and mood, impacting eating behaviors and emotional responses to food. The lateral hypothalamus is involved in arousal, motivation, and energy levels, with connections to depression. Lastly, the ventromedial nucleus helps regulate emotional responses and aggression, influencing anxiety and depression. These regions work together to regulate stress, emotions, and behavior, making them integral to mental health. Dysregulation of this axis is commonly observed in anxiety, depression, and PTSD, exacerbating symptoms by maintaining a heightened stress state. The basal ganglia, involved in motor control, motivation, and reward processing, also contribute to mood regulation, with dysfunctions in this region observed in conditions such as depression and OCD. Understanding these brain regions and their roles in mental health informs the development of targeted treatments, including medications that address neurotransmitter imbalances and therapeutic interventions designed to improve brain function. This neurobiological perspective underscores the complexity of mental health disorders and highlights the importance of advancing treatments that address the underlying mechanisms within these critical brain areas [16].

Genetic predispositions and family history of mental disorders

Genetic predispositions and family history play significant roles in the development of mental disorders, indicating that these conditions often have a hereditary component. While environmental factors, lifestyle choices, and social influences also contribute, understanding the genetic underpinnings provides insight into the complexity of mental health.

Genetic Predispositions

Research indicates that mental disorders often run in families, suggesting a genetic basis. For instance, studies estimate the heritability of conditions such as schizophrenia to be approximately 80%, while MDD has a heritability estimate of approximately 37% [17]. This implies that genetics account for a substantial portion of the risk of developing these disorders. Specific genes have been associated with various mental health conditions. For example, variations in the serotonin transporter gene (*5-HTTLPR*) are linked to depression and anxiety disorders. Other genes involved in dopamine regulation, such as those affecting the D2 receptor, have been implicated in schizophrenia and bipolar disorder. Advances in genomic research have led to the development of polygenic risk scores, which aggregate the effects of multiple genetic variants to estimate an individual's risk for certain mental disorders. This approach highlights that mental health is influenced by the cumulative effect of many genes, each contributing a small risk [17].

Family History

Individuals with a family history of mental disorders are at a higher risk of developing similar conditions. For example, a person with a first-degree relative (parent or sibling) diagnosed with bipolar disorder has a significantly increased risk compared to the general population. The risk is also higher for other disorders, such as depression, anxiety, and substance use disorders. Family history not only reflects genetic predispositions but also encompasses shared environmental factors. Families often provide similar environments, experiences, and stressors, which can contribute to the onset of mental health issues. For example, exposure to trauma, socioeconomic challenges, or parental mental health problems can influence the mental well-being of family members. The field of epigenetics explores how environmental factors can affect gene expression without altering the DNA sequence itself. This means that family history may influence mental health through epigenetic changes, where adverse experiences can modify gene activity across generations, potentially increasing the risk for descendants [18].

Implications for Treatment and Prevention

Understanding genetic predispositions and family history can inform personalized approaches to mental health treatment and prevention. For individuals with a family history of mental disorders, early intervention strategies, such as therapy and lifestyle modifications, may be beneficial. Genetic counseling can also provide insights into risk factors and guide decisions regarding treatment options. While genetics play a crucial role in mental health, it is essential to consider the interplay between genetic predispositions and environmental factors. Ongoing research in genomics and epigenetics will continue to shed light on the complexities of mental disorders, paving the way for more targeted interventions and preventive strategies [19].

Emerging lifestyle factors affecting mental well-being

Sleep Patterns and Their Impact on Mental Health

Sleep patterns are intricately linked to mental health, with a bidirectional relationship that underscores their mutual influence. Poor sleep, whether due to insufficient duration or disrupted quality, can exacerbate mental health issues such as depression, anxiety, and bipolar disorder. Sleep deprivation affects the brain's emotional regulation, increasing sensitivity to stress and impairing decision-making and memory. Chronic sleep problems are also associated with a heightened risk of developing mental health disorders. Conversely, mental health conditions often disrupt sleep patterns, leading to insomnia, hypersomnia, or fragmented sleep. For instance, anxiety can cause difficulty falling asleep, while depression may lead to early waking or excessive sleep. Sleep disturbances also impair the body's ability to recover and regulate neurotransmitters, further deepening the cycle of poor mental health. Adopting healthy sleep habits, such as maintaining a consistent sleep schedule, creating a calming bedtime routine, and managing stress, is crucial for supporting mental well-being [20].

Impact of Sleep Patterns on Mental Health

Adequate sleep is crucial for cognitive function, emotional regulation, and overall mental health. Research shows that individuals who consistently obtain insufficient sleep (typically defined as less than seven to eight hours per night) are at a higher risk for developing mental health disorders such as anxiety and depression. Conversely, good sleep quality characterized by restful, uninterrupted sleep is associated with improved mood and emotional resilience. The body’s internal clock, or circadian rhythm, regulates sleep-wake cycles and influences various physiological processes, including hormone release and brain function. Disruptions to circadian rhythms, often caused by irregular sleep patterns, shift work, or excessive screen time, can lead to mood disturbances and heightened susceptibility to mental health issues. For instance, misalignment of circadian rhythms is linked to increased rates of depression and bipolar disorder. Conditions such as insomnia, sleep apnea, and restless leg syndrome can have a direct negative impact on mental health. Insomnia is frequently associated with anxiety disorders and depression, creating a vicious cycle where poor sleep worsens mental health, leading to further sleep disturbances. Sleep apnea, characterized by repeated interruptions in breathing during sleep, has also been linked to mood disorders, cognitive decline, and increased risk of suicidal ideation. Quality sleep is vital for emotional regulation and resilience. Sleep deprivation can impair the brain's ability to process emotions and manage stress, leading to increased irritability, anxiety, and vulnerability to mood swings. Studies have shown that individuals who experience chronic sleep deprivation are more likely to exhibit symptoms of anxiety and depression. Sleep influences the balance of key neurotransmitters involved in mood regulation, such as serotonin and dopamine. Sleep disruptions can alter the levels of these neurotransmitters, contributing to mood disorders. For example, reduced serotonin levels due to inadequate sleep may exacerbate feelings of sadness and anxiety [20].

Strategies to Improve Sleep and Mental Health

Improving sleep quality is essential for enhancing mental health and overall well-being, as sleep and mental health are deeply interconnected. Adopting specific strategies can significantly improve both. Establishing a consistent sleep schedule by going to bed and waking up at the same time daily helps regulate circadian rhythms, promoting more restorative sleep. Creating a relaxing bedtime routine with calming activities such as reading, meditation, or gentle stretching can signal the body to prepare for sleep. Limiting screen time, particularly reducing exposure to blue light from devices at least an hour before bed, supports melatonin production and reduces sleep disruptions. Regular physical activity is another effective strategy, as exercise improves sleep quality and alleviates symptoms of anxiety and depression, fostering a healthier mind and body. Optimizing the sleep environment is equally important ensuring the bedroom is quiet, dark, and maintained at a comfortable temperature creates an atmosphere conducive to restful sleep. Recognizing the significant impact of sleep patterns on mental health highlights the importance of addressing sleep issues and prioritizing healthy sleep habits. By integrating these practices into daily life, individuals can improve their mental health outcomes, reduce stress, and enhance their overall quality of life. These strategies underscore the importance of a holistic approach to mental well-being, where improving sleep serves as a foundational step toward greater resilience and emotional balance [21].

Diet, exercise, and the role of physical health in psychological well-being

The interplay between physical health and psychological well-being is increasingly recognized as crucial for overall mental health. Key lifestyle factors, such as diet and exercise, significantly impact mental well-being, influencing mood, cognitive function, and resilience against stress and mental health disorders.

The Role of Diet in Psychological Well-Being

Emerging research in nutritional psychology examines how dietary choices influence mental health. A balanced diet rich in essential nutrients is essential for optimal brain function and emotional regulation. Nutrients such as omega-3 fatty acids, vitamins (especially B vitamins), minerals (such as magnesium and zinc), and antioxidants are linked to reduced symptoms of depression and anxiety. A balanced diet plays a significant role in psychological well-being, with certain nutrients having a direct impact on brain health. For omega-3 fatty acids, crucial for brain function and mood regulation, examples include fatty fish (salmon, mackerel, sardines), flaxseeds, chia seeds, and walnuts. Vitamins also play a vital role; B vitamins like B6, B12, and folate, found in leafy greens, whole grains, eggs, and legumes, are essential for neurotransmitter function and mental clarity. Vitamin D, which influences mood and cognitive function, can be sourced from sunlight, fortified dairy products, and fatty fish. For minerals, magnesium, found in spinach, almonds, and pumpkin seeds, is known for its calming effect, while zinc, present in pumpkin seeds, shellfish, and beans, supports cognitive function. Antioxidants, which protect the brain from oxidative stress, can be found in berries (blueberries, strawberries), dark chocolate, and green tea and are key for reducing inflammation and supporting mental health. By incorporating these nutrient-rich foods, individuals can promote better psychological well-being [22].

The gut microbiome plays a significant role in mental health through the gut-brain axis, a bidirectional communication system between the gastrointestinal tract and the brain. A diet high in processed foods, sugars, and unhealthy fats can negatively affect gut microbiota diversity, leading to dysbiosis, which is associated with mood disorders. Conversely, a diet rich in fruits, vegetables, whole grains, and fermented foods can promote a healthy gut microbiome and support mental well-being. Chronic inflammation is increasingly recognized as a contributing factor to mental health disorders. Diets high in processed foods can promote inflammation, while anti-inflammatory foods (such as fatty fish, nuts, seeds, and leafy greens) can help reduce inflammation and improve mood. Studies suggest that adopting an anti-inflammatory diet may reduce the risk of developing depression and anxiety [22].

The Role of Exercise in Psychological Well-Being

Regular exercise has been shown to improve mood and alleviate symptoms of depression and anxiety. Physical activity stimulates the release of endorphins and other neurotransmitters, such as serotonin and dopamine, which enhance feelings of well-being and reduce stress. Even moderate-intensity exercise, such as walking or cycling, can have significant benefits for mental health. Exercise serves as a powerful tool for stress management. Engaging in physical activity helps lower cortisol levels (the body's primary stress hormone) and promotes relaxation. Activities like yoga and tai chi not only improve physical fitness but also incorporate mindfulness and breathing techniques that enhance emotional resilience. Yoga and tai chi are mind-body practices that offer significant benefits for both physical and mental health. These exercises combine physical postures, breathing techniques, and mindfulness, promoting relaxation, flexibility, and strength. In yoga, various asanas (poses) are performed with a focus on controlled breathing (pranayama) and meditation, helping to reduce stress, anxiety, and depression. The deep, conscious breathing involved in yoga activates the parasympathetic nervous system, reducing the body's stress response and promoting a sense of calm. Tai chi, a Chinese martial art, involves slow, flowing movements and deep breathing, emphasizing balance, coordination, and mental focus. The practice has been shown to reduce stress, enhance mood, and improve cognitive function by integrating mindfulness into movement. Both practices encourage emotional resilience by helping individuals stay present, manage negative emotions, and build a sense of inner peace. Regular physical activity is associated with improved cognitive function and memory. Studies show that exercise increases blood flow to the brain, promoting neurogenesis (the growth of new neurons) and enhancing cognitive performance. This is particularly important for mental health, as cognitive decline is often linked to mood disorders. Group exercise activities can foster social connections, which are crucial for psychological well-being. Engaging in physical activities with others provides social support, reduces feelings of isolation, and enhances overall mood [23].

Integrating Diet and Exercise for Better Mental Health

To optimize psychological well-being, it is essential to adopt a holistic approach that combines a balanced diet with regular physical activity. Here are some strategies to enhance both physical and mental health. Incorporate a variety of nutrient-dense foods into the diet, including fruits, vegetables, whole grains, lean proteins, and healthy fats. Consider consulting a nutritionist for personalized dietary guidance. Aim for at least 150 minutes of moderate aerobic exercise or 75 minutes of vigorous exercise each week, along with strength training exercises at least twice a week. Practice mindfulness by being aware of hunger cues, making conscious food choices, and paying attention to the sensations experienced during physical activity. Establish achievable dietary and exercise goals to create a sense of accomplishment and promote long-term adherence to healthy habits. Diet, exercise, and overall physical health play crucial roles in psychological well-being. By prioritizing healthy lifestyle choices, individuals can significantly enhance their mental health, resilience, and quality of life [24].

Technological overuse and its psychological consequences (screen time, social media)

Technological overuse, particularly through excessive screen time and social media engagement, has become a prominent concern in modern society. The proliferation of digital devices and platforms has transformed how individuals interact, work, and seek information, but this has also led to various psychological consequences that can impact mental health and well-being.

Psychological Consequences of Technological Overuse

Research indicates that excessive screen time, particularly on social media, is associated with heightened levels of anxiety and depression. Constant exposure to curated online personas can lead to feelings of inadequacy, low self-esteem, and social comparison, which contribute to emotional distress. Studies have shown that individuals who spend more time on social media report greater feelings of loneliness and depressive symptoms. Ironically, while social media is designed to connect people, excessive use can lead to social isolation. Individuals may substitute online interactions for face-to-face relationships, resulting in weakened social bonds and increased feelings of loneliness. The quality of social interactions can also suffer, as online communication often lacks the emotional richness of in-person conversations. Excessive screen time, especially before bedtime, can disrupt sleep patterns due to the blue light emitted by screens, which interferes with melatonin production. Poor sleep quality is closely linked to mental health issues, including anxiety and depression. Sleep disturbances can also exacerbate existing psychological conditions, creating a vicious cycle of poor mental health and inadequate sleep. The fast-paced nature of digital media can impair attention spans and cognitive function. Frequent notifications and the habit of multitasking while using technology can lead to diminished focus and productivity. Studies have shown that individuals who engage in heavy media consumption may experience difficulties in maintaining attention and may struggle with tasks requiring sustained cognitive effort. Technological overuse can lead to addictive behaviors, where individuals feel compelled to engage with their devices or social media platforms, even when it interferes with daily life or responsibilities. This compulsive use can lead to increased anxiety when individuals are unable to access their devices or when they are offline. Social media platforms often promote unrealistic standards of beauty and success, leading to body image dissatisfaction and eating disorders, particularly among adolescents and young adults. The constant exposure to idealized images can foster negative self-perception and increase vulnerability to mental health issues related to body image [25,26].

Mitigating the Psychological Consequences

To address the psychological consequences of technological overuse, individuals can adopt several strategies. Implementing regular breaks from technology can help reduce dependency and promote healthier habits. Designating specific times for device-free activities can encourage more meaningful face-to-face interactions and help restore balance. Practicing mindfulness in technology usage involves being aware of the time spent on screens and the emotional responses triggered by social media interactions. Setting intentional limits on screen time can foster a healthier relationship with technology. Focus on meaningful connections rather than the quantity of online interactions. Prioritizing in-person relationships and engaging in activities that promote social bonds can mitigate feelings of isolation. Establishing a bedtime routine that includes limiting screen time to at least an hour before sleep can improve sleep quality. Creating a calming environment can also promote better rest. Engaging in physical activity and hobbies can provide a healthy distraction from screens and enhance overall well-being. Pursuing offline interests can also foster creativity and personal fulfillment. While technology offers numerous benefits, its overuse can have significant psychological consequences. By recognizing the impacts of excessive screen time and social media engagement and implementing strategies to mitigate these effects, individuals can enhance their mental health and overall quality of life [27].

Preventive approaches to mental health

Preventive approaches to mental health are essential for promoting psychological well-being, reducing the incidence of mental health disorders, and enhancing overall quality of life. By focusing on early intervention, education, and supportive environments, preventive strategies can effectively address risk factors and foster resilience in individuals and communities. Here are key preventive approaches to mental health:

Education and Awareness

Education and awareness are fundamental to improving mental health outcomes and reducing the stigma that often prevents individuals from seeking help. By increasing understanding of mental health issues, educational programs can empower individuals to recognize symptoms early, seek appropriate support, and adopt healthy coping strategies. These programs can demystify mental health disorders, emphasizing that they are treatable conditions rather than personal weaknesses, fostering a more accepting and supportive environment. Workshops and training sessions in schools, workplaces, and communities play a pivotal role in promoting mental health literacy. In schools, such programs can teach students about stress management, emotional regulation, and resilience-building techniques, equipping them with skills to navigate challenges effectively. In workplaces, mental health workshops can raise awareness about the impact of stress and burnout while providing employees with tools to maintain work-life balance and access resources when needed. Community-based initiatives can further extend these benefits by reaching diverse populations, addressing cultural barriers, and creating support networks. Such educational efforts also contribute to early intervention, as informed individuals are more likely to identify warning signs in themselves or others and take proactive steps. Integrating mental health education into broader health campaigns and policies ensures sustainability and reach. By fostering a culture of understanding and openness, education and awareness initiatives pave the way for improved mental well-being, reduced stigma, and stronger, more resilient communities [28].

Early Intervention Programs

Early intervention programs are essential for identifying and addressing mental health issues before they escalate, promoting better long-term outcomes. Regular mental health screenings in schools and primary care settings are a critical component of these programs, enabling the early identification of individuals at risk for mental health disorders. By recognizing warning signs early, healthcare providers, educators, and families can implement timely interventions, reducing the severity and duration of mental health challenges. Schools play a pivotal role in early intervention by offering programs that address specific risk factors such as bullying, academic pressure, and social isolation. Bullying prevention initiatives, for example, foster a safe and supportive environment, reducing the likelihood of anxiety, depression, and other related issues. Stress management training in workplaces can similarly mitigate the impact of chronic stress, preventing burnout and promoting emotional well-being among employees. Community-based early intervention programs further enhance these efforts by providing accessible resources and support networks tailored to local needs. These programs often include counseling services, peer support groups, and educational workshops aimed at building resilience and coping skills. By prioritizing early detection and targeted interventions, these programs not only reduce the burden of mental health disorders but also empower individuals to lead healthier, more fulfilling lives [29].

Promotion of Healthy Lifestyles

Promoting healthy lifestyles is a key strategy for enhancing mental well-being and preventing mental health disorders. Regular physical activity is a well-documented preventive measure, as exercise stimulates the release of endorphins, which improve mood, reduce stress, and enhance overall emotional resilience. Beyond its physiological benefits, physical activity fosters a sense of achievement and social connection, particularly when done in group settings. Community programs that offer access to recreational activities, fitness classes, and outdoor spaces can make exercise more accessible and appealing to diverse populations. Healthy eating habits also play a crucial role in mental health. Nutritional education initiatives can raise awareness about the connection between diet and mental well-being, emphasizing the importance of consuming nutrient-rich foods that support brain function. Diets rich in omega-3 fatty acids, vitamins, and minerals have been linked to reduced risks of depression and anxiety. Encouraging the consumption of whole grains, fruits, vegetables, and lean proteins while limiting processed foods can provide the necessary nutrients for optimal brain health. By integrating physical activity and nutrition education into schools, workplaces, and community settings, these initiatives can promote sustainable, healthy habits. Such programs not only improve mental health outcomes but also contribute to overall physical health, creating a foundation for a healthier, more resilient society [30].

Mindfulness and Stress Reduction Techniques

Mindfulness and stress reduction techniques are powerful tools for promoting mental well-being and resilience. Programs that incorporate mindfulness practices, such as meditation, yoga, and deep-breathing exercises, help individuals cultivate present-moment awareness, improve emotional regulation, and manage stress more effectively. These practices have been scientifically proven to reduce symptoms of anxiety and depression by calming the nervous system and fostering a sense of inner balance. Mindfulness training not only enhances self-awareness but also equips individuals with skills to respond to stressors in healthier ways. It encourages a non-judgmental attitude toward thoughts and emotions, reducing reactivity and promoting a sense of control. Yoga, in particular, combines physical movement with mindfulness, offering both physical and mental health benefits. Teaching additional coping strategies, such as problem-solving skills and emotional regulation techniques, further empowers individuals to navigate life’s challenges. These strategies help build resilience by enabling individuals to identify stressors, develop constructive responses, and maintain a balanced perspective during difficult situations. By integrating mindfulness and stress reduction programs into schools, workplaces, and community centers, individuals can gain lifelong tools for managing stress and enhancing mental health. Such initiatives not only reduce vulnerability to mental health disorders but also foster a culture of emotional well-being and resilience [31].

Social Support and Community Engagement

Social support and community engagement are essential for maintaining mental well-being and fostering resilience. Strong, supportive relationships and social networks provide individuals with emotional comfort, a sense of belonging, and practical assistance during difficult times. Community programs that encourage social interactions, such as support groups, peer mentoring, and group activities, help individuals build meaningful connections, combat isolation, and share experiences. These social ties are protective factors that can buffer against stress and reduce the risk of developing mental health disorders. Encouraging participation in community service or volunteer work can further enhance mental well-being by promoting a sense of purpose and fulfillment. Volunteering not only allows individuals to contribute to their communities but also helps them develop social bonds and increase self-esteem. Research has shown that those who engage in acts of kindness and community involvement experience improved mood, reduced feelings of loneliness, and lower levels of anxiety and depression. By fostering environments that prioritize social support and community engagement, individuals are more likely to feel valued and connected. These programs create spaces where people can share resources, seek help when needed, and strengthen their emotional resilience. As a result, they play a vital role in preventing mental health issues and promoting overall well-being [32].

Workplace Mental Health Initiatives

Workplace mental health initiatives are vital for fostering a healthy and supportive environment. Providing access to mental health resources, such as counseling services and employee assistance programs (EAPs), allows employees to proactively manage stress and address mental health concerns. Implementing policies that promote work-life balance, such as flexible work hours, remote work options, and designated mental health days, can significantly reduce burnout and enhance well-being. Encouraging open communication about mental health without stigma is essential. Regular workshops on stress management, mindfulness, and resilience can equip employees with tools to cope with challenges. Training managers to recognize signs of mental health issues ensures timely support and intervention. Fostering a culture of empathy and understanding contributes to increased productivity, higher employee morale, and better retention rates. Investing in mental health initiatives not only benefits individual employees but also strengthens the organization’s overall performance by promoting a positive and balanced work environment [33].

Access to Mental Health Resources

Access to mental health resources is essential for prevention and early intervention. Providing therapy and counseling services can help individuals manage stress, anxiety, and other mental health challenges before they escalate. There are several public domain services and websites where individuals suffering from mental health issues can access support. The National Suicide Prevention Lifeline (USA) offers 24/7 crisis intervention and can be reached at 988. SAMHSA (Substance Abuse and Mental Health Services Administration) provides a national helpline for individuals seeking treatment and support, available at 1-800-662-HELP. Crisis Text Line offers free, confidential text support for people in crisis, simply by texting HELLO to 741741. Mental Health America (MHA) provides resources and tools for individuals to find support in their local area through their website. For global access, 7 Cups offers free online therapy and peer support, while Better-Help and Talk-space provide affordable online therapy services. Mind (UK) and Beyond Blue (Australia) offer mental health support services, including helplines, chat services, and community programs. These platforms and services play a crucial role in ensuring individuals can seek help at any time, from anywhere, fostering better mental health awareness, prevention, and early intervention [34]. Telehealth options, including online therapy and virtual support groups, play a vital role in increasing accessibility, especially for individuals in remote or underserved areas who may have limited access to in-person care. Establishing community-based mental health services is equally important. These centers can offer a range of support, including crisis intervention, mental health education, and referral services, ensuring that help is available at the local level. Creating a culture that prioritizes mental well-being through accessible services fosters empathy, reduces stigma, and promotes early help-seeking behaviors. By expanding access to mental health resources, we can empower individuals to take proactive steps toward mental wellness, improving overall public health and creating stronger, more resilient communities [34].

Policy and advocacy

Supporting policies that promote mental health awareness, funding for mental health services, and protections for individuals with mental health disorders can create a more supportive environment for mental well-being. Encouraging the integration of mental health services within primary healthcare settings can facilitate early intervention and reduce barriers to accessing care. Preventive approaches to mental health are essential for fostering resilience, reducing the incidence of mental health disorders, and enhancing overall well-being. By prioritizing education, early intervention, healthy lifestyles, social support, and access to resources, individuals and communities can create environments that promote mental health and support those in need. Ultimately, investing in prevention is key to building a healthier and more resilient society [35].

Challenges in mental health care access

Accessing mental health care remains a significant challenge for many individuals, leading to unmet needs and exacerbated mental health issues. The societal stigma surrounding mental health often discourages individuals from seeking help. Fear of judgment can prevent people from discussing their struggles or pursuing treatment, leaving many without the necessary support. In many regions, particularly rural and underserved urban areas, there is a shortage of mental health professionals. Long wait times for appointments and a lack of specialized services further exacerbate access issues. The cost of mental health care can be prohibitive, especially for those without insurance or with inadequate coverage. For many, especially those living in remote areas, transportation challenges can hinder access to care. Inadequate public transport options or lack of personal vehicles can prevent individuals from reaching mental health facilities. Navigating mental health services can be daunting, with varying levels of care, referral processes, and administrative hurdles that complicate access for those in need [36].

Future directions in mental health research and interventions

The field of mental health research and interventions is evolving rapidly, driven by advancements in technology, increased awareness of mental health issues, and a growing understanding of the complex factors influencing psychological well-being. Here are several key future directions in mental health research and interventions:

Integration of Technology in Mental Health Care

The integration of technology into mental health care has revolutionized how services are delivered, improving accessibility and patient engagement. Teletherapy, in particular, has expanded access to mental health services, especially in underserved or remote areas where traditional care options are limited. This virtual care model has proven invaluable in breaking geographical and logistical barriers, offering a lifeline to individuals who might otherwise struggle to access therapy. Future research will likely focus on optimizing these virtual care models, assessing their effectiveness compared to in-person treatments, and integrating them seamlessly into existing healthcare systems. This includes developing guidelines for best practices, ensuring patient confidentiality, and addressing disparities in digital access. The rise of mental health apps further complements these efforts by providing tools for self-monitoring, cognitive-behavioral therapy (CBT), mindfulness practices, and mood tracking. These apps empower individuals to actively participate in their mental health management, enhancing treatment adherence and fostering a sense of control. Features like real-time feedback, reminders, and progress tracking make these tools valuable adjuncts to professional care. As technology continues to evolve, integrating AI-driven insights, virtual reality (VR), and wearable devices could further enhance mental health interventions. By combining these innovations with traditional methods, the future of mental health care promises to be more accessible, personalized, and effective [37].

Personalized and Precision Mental Health

The future of mental health care lies in personalized and precision approaches, which aim to tailor treatments to individual needs by integrating biological, psychological, and social factors. Emerging research is increasingly focused on identifying biomarkers such as genetic markers, neuroimaging patterns, and hormonal levels that can predict mental health disorders or their progression. Understanding genetic predispositions and their interaction with environmental influences will enable clinicians to design interventions that are more effective and individualized. Big data analytics and AI are poised to revolutionize this field by analyzing vast datasets to uncover patterns and trends in mental health. These technologies can identify population-specific risk factors, predict treatment outcomes, and optimize resource allocation. For example, AI-driven tools could help clinicians recommend therapies based on a patient’s unique genetic, neurobiological, and lifestyle profiles, improving treatment efficacy and reducing trial-and-error approaches. Personalized mental health care also considers psychological and social factors, integrating them with biological insights to create holistic treatment plans. For instance, combining CBT with pharmacological treatments tailored to an individual's genetic makeup could enhance outcomes. By advancing personalized and precision mental health, future interventions will not only address individual needs more effectively but also improve overall mental health trends, fostering resilience and recovery [38].

Focus on Preventive Approaches

Future mental health strategies are expected to prioritize preventive approaches, focusing on early intervention programs designed for at-risk populations, particularly children and adolescents. Early interventions can address risk factors such as trauma, stress, and social isolation before they escalate into severe mental health disorders. By identifying and supporting vulnerable individuals early, these programs aim to reduce the prevalence and impact of conditions such as anxiety, depression, and behavioral disorders. Community support systems will play a pivotal role in these preventive efforts. Strengthening social networks and providing accessible resources within communities can foster resilience and promote mental well-being. Programs emphasizing mental health awareness and education will empower individuals to recognize early signs of distress and seek help proactively. Additionally, fostering social connections through community initiatives can mitigate feelings of isolation and create environments that support emotional health. Innovative approaches may include integrating mental health education into school curricula, providing training for parents and teachers to identify warning signs, and expanding access to counseling and support services. Digital platforms and telehealth services may also enhance outreach, making mental health resources more accessible. By focusing on prevention, future interventions aim to reduce the long-term societal and economic burden of mental health disorders while promoting healthier, more resilient communities [39].

Understanding Social Determinants of Mental Health

Understanding the social determinants of mental health is critical for developing comprehensive approaches to improve mental health outcomes. Social determinants, including socioeconomic status, education, employment, housing, and community environment, profoundly influence mental health and well-being. For instance, individuals from lower socioeconomic backgrounds often face chronic stress, financial instability, and limited access to healthcare, increasing their vulnerability to mental health disorders such as depression and anxiety. Similarly, inadequate education can restrict opportunities for stable employment, further perpetuating stress and poor mental health. The community environment also plays a significant role, as supportive social networks and safe neighborhoods promote resilience, while isolation, discrimination, and exposure to violence exacerbate mental health challenges. Addressing these determinants requires a shift from symptom-focused treatment to strategies that target root causes. This involves improving access to quality education, ensuring equitable health care, creating economic opportunities, and fostering inclusive, supportive communities. Future research should prioritize understanding the interplay between these factors and their cumulative impact on mental health. Collaborative efforts among psychologists, sociologists, public health experts, and policymakers are essential to design and implement holistic interventions. Such collaboration can inform policies that reduce inequalities, enhance social support systems, and promote mental health literacy. By addressing the social determinants of mental health, we can move toward sustainable solutions that improve overall well-being and reduce the burden of mental health disorders on individuals and society. This integrated approach emphasizes prevention and resilience, creating a foundation for healthier communities [40].

Culturally Sensitive Interventions

Future research will emphasize the importance of culturally sensitive approaches to mental health care, recognizing the diverse backgrounds and experiences of individuals. This includes tailoring interventions to fit cultural beliefs and practices. Expanding mental health research to include global perspectives can inform the development of effective interventions across different cultural and socioeconomic contexts. The resurgence of interest in psychedelics for therapeutic use has prompted research into their efficacy in treating various mental health disorders, such as PTSD, depression, and anxiety. Future studies will explore the safety, mechanisms, and long-term effects of these substances. Advancements in neuroscience can provide insights into the biological underpinnings of mental health disorders, guiding the development of novel interventions targeting specific brain regions and neurotransmitter systems [41].

Improving Access to Mental Health Services

Future directions in mental health research will likely focus on advocating for policies that enhance access to care, reduce stigma, and increase funding for mental health services. Research will explore effective workplace interventions to promote mental well-being among employees, such as stress management programs and mental health days. The future of mental health research and interventions holds great promise as it adapts to the evolving needs of individuals and communities. By embracing innovative technologies, personalized approaches, and a comprehensive understanding of the factors influencing mental health, researchers and practitioners can work toward creating effective and accessible mental health care systems that promote well-being for all.

**Figure 1:**
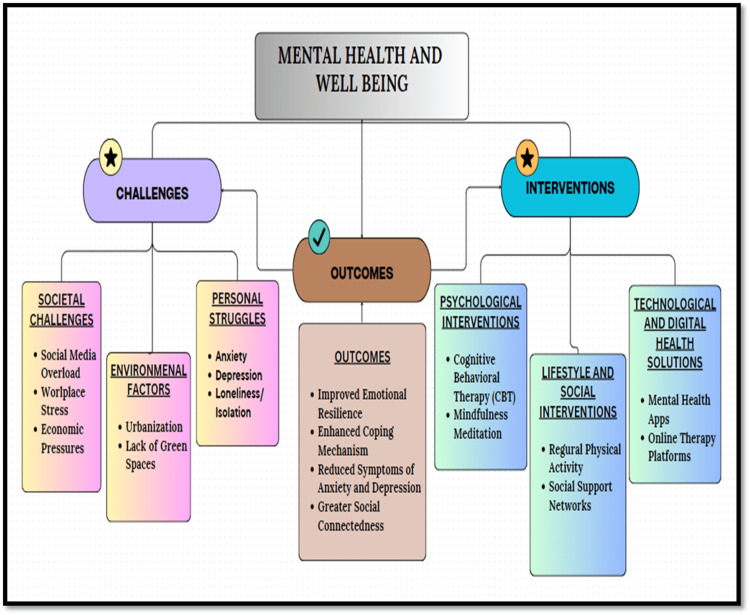
Mental health and well-being in the modern era, highlighting the key challenges and interventions Challenges include digital overload, leading to stress from constant connectivity; social isolation, resulting from reduced face-to-face interactions; workplace stress, stemming from high demands and low support; economic pressure, which induces anxiety due to financial instability; and sleep disruption, affecting overall health. Interventions involve mindfulness practices to manage stress, work-life balance policies for improved employee well-being, community support to combat isolation, therapy and counseling for professional help, and telehealth services for accessible mental health care. Image credit: Dr. Ghizal Fatima.

Figure 1 presents the key challenges, outcomes, and interventions for mental health and well-being in the modern era.

## Conclusions

The landscape of mental health care and research is at a pivotal juncture, marked by significant advancements and emerging challenges. However, critical gaps remain in our understanding of the underlying mechanisms of mental health disorders, particularly how disruptions in mental health can lead to long-term effects on individuals and communities. The effects of decreased mental health are far-reaching, influencing not only emotional well-being but also physical health, productivity, and overall quality of life. Chronic mental health issues, if left unaddressed, can lead to a cascade of problems such as increased risk of substance abuse, social isolation, and higher mortality rates. Coping mechanisms, such as maladaptive behaviors and lack of social support, can exacerbate these effects, making it essential to focus on both prevention and early intervention.

To overcome these challenges, it is imperative to prioritize a systematic, multi-pronged approach that includes early screening, personalized treatment plans, and increased access to care, especially in underserved communities. Future research should focus on understanding the neurobiological, psychological, and social factors that contribute to mental health disorders while also exploring the efficacy of innovative treatments, including digital mental health tools and teletherapy. Policymakers must advocate for mental health education, public awareness campaigns, and policies that reduce stigma and encourage early help-seeking behaviors. By fostering a culture of mental well-being, integrating mental health care into primary health services, and ensuring culturally sensitive approaches, we can enhance the accessibility and effectiveness of interventions. Ultimately, these efforts will help build resilience, reduce the long-term impacts of mental health issues, and improve the overall quality of life for individuals and communities worldwide.
